# Psychological impact of COVID-19 and determinants among Spanish university students

**DOI:** 10.3389/fpubh.2023.1252849

**Published:** 2023-10-18

**Authors:** Jesús Cebrino, Silvia Portero de la Cruz

**Affiliations:** ^1^Department of Preventive Medicine and Public Health, University of Seville, Seville, Spain; ^2^Department of Nursing, Pharmacology and Physiotherapy, University of Córdoba, Córdoba, Spain; ^3^Research Group GE10 Clinical and Epidemiological Research in Primary Care, Instituto Maimónides de Investigación Biomédica de Córdoba (IMIBIC), Hospital Universitario Reina Sofía, Córdoba, Spain

**Keywords:** COVID-19, fear, public health, Spain, students

## Abstract

**Background:**

University students are a vulnerable population and faced a significant psychological impact from the COVID-19 pandemic. Therefore, this study aimed to determine the level of fear of COVID-19 among university students and to evaluate the possible relationship between fear of COVID-19 and socio-demographic, health-related determinants, variables related to the COVID-19 and variables related to the psychological impact of the COVID-19 pandemic.

**Methods:**

We conducted a cross-sectional study between December 2020 and December 2021 on a sample of 950 university students from two universities in southern Spain. Participants completed a form that collected socio-demographic, health-related and COVID-related variables, a validated questionnaire related to the psychological impact of the COVID-19 pandemic and the fear of COVID Scale (FCV-19S). Descriptive, inferential, and multivariable linear regression analyzes were conducted.

**Results:**

The mean FCV-19S score was 14.86 ± 5.16 points. The factors identified as predictors of FCV-19S were being female (*p* < 0.001), holding religious beliefs (*p* = 0.04), living in towns with over 10,000 inhabitants (*p* < 0.01), living with someone vulnerable to COVID-19 (*p* = 0.02), watching TV to keep informed about COVID-19 (*p* < 0.01), believing in a low probability of surviving if infected with COVID-19 (*p* < 0.001), having a higher level of death anxiety (*p* < 0.001) and suffering from insomnia (*p* < 0.001).

**Conclusion:**

An average fear of COVID-19 score of 14.86 ± 5.16 points has been found among university students in Spain. These findings can aid in identifying specific factors contributing to fear of COVID-19 and in developing coping strategies to alleviate the stress of the pandemic.

## 1. Introduction

Severe acute respiratory syndrome coronavirus 2 (SARS-CoV-2) was identified as the cause of coronavirus disease 19 (COVID-19) in December 2019 (1). The rapid person-to-person transmission, as well as the severity of the virus across the globe (2), led to the implementation of several measures in Spain, including a general lockdown that started on March 15, 2020, and concluded on June 21, 2020. During this period of confinement, comprehensive measures were implemented, including the closure of restaurants, schools, universities, and workplaces which involved close physical contact. Governments also enforced physical distancing through quarantines, travel restrictions, and closures of various establishments such as theaters, cinemas, museums, and stadia. Remote work and online education became widespread, while the limited number of activities permitted included essential work, grocery shopping, caregiving, and medical visits (3). Nevertheless, during those 100 days of lockdown, there were 245,938 total confirmed cases of infection and 28,322 total deaths in Spain (4).

The lockdown period led not only a radical change in the population’s lifestyle (5), but also fear of COVID-19 (6) and a negative effect on mental health (7, 8) with reports of symptoms of common mental disorders in 35–50% of the population (9, 10). Fear, defined as an adaptive response to real or abstract threats which is crucial for survival (11), stands out as one of the most prevalent psychological reactions to pandemic diseases, and differs in responses to other disasters (12).

A multitude of adverse conditions, such as social isolation, uncertainty, chronic illness, financial hardships, disruptions to daily routines, and the loss of family members to COVID-19, particularly among the older adult, contributed to heightened levels of fear during the pandemic (13–16). This pervasive fear has had a profound and widespread impact on the mental health of millions of individuals worldwide (17). Several studies have investigated this impact on different groups (18–22), including university students, who were at risk due to the continued spread of the pandemic, strict isolation measures, and the restrictions on interpersonal relationships (23, 24).

In Spain, the level of fear of COVID-19 among university students (25) is higher than in other countries such as Turkey (26), China (27), Pakistan (28) or Russia and Belarus (29). Multiple studies worldwide performed in that population (30, 31) have shown the impact that fear of COVID-19 has on several psychological constructs. Thus, on the one hand, fear of COVID-19 plays a pivotal role in the initiation and progression of sleep difficulties, stress, panic and common mental disorders (32–37). On the other hand, death anxiety appears as an abnormal response when individuals experience fear regarding COVID-19 (38, 39). Additionally, previous studies reveal that other constructs such as social support, optimism, subjective happiness and resilience mitigates the fear of COVID-19, fostering better mental health in the battle against the virus (40–42).

Additionally, it is important to consider that the fear of COVID-19 has had an impact on the lifestyle habits of university students, such as resorting to increased alcohol or tobacco consumption as coping responses to the stressors associated with the COVID-19 pandemic (43), adopting less healthy dietary practices (44), and exhibiting a low level of physical activity (45).

The COVID-19 pandemic continues to pose significant health challenges (46, 47) and exerts an ongoing impact on a wide range of psychological responses in individuals (48), including university students (49). Despite the growing number of studies on the fear of COVID-19 worldwide, there is a noticeable gap in the scientific literature, to the best of our knowledge, particularly in terms of concurrently evaluating a variety of psychological constructs and assessing their influence in relation to the fear of COVID-19 among university students in Spain. Therefore, the present study aims to determine the level of fear of COVID-19 among university students and to evaluate the possible relationship between fear of COVID-19 and socio-demographic/health-related determinants, variables related to the COVID-19 and variables related to the psychological impact of the COVID-19 pandemic.

## 2. Materials and methods

### 2.1. Participants and procedure

We conducted a cross-sectional study among Spanish university students from December 2020 to December 2021.

The study was carried out in 12 different bachelor’s degree courses at two public universities located in the southern region of Spain, Andalusia. The majority of the students were taking degree courses in Health Sciences. Public university 1 had a total of 6,965 university students taking nine different bachelor’s degrees, while public university 2 contained a total of 1,417 university students studying for three different bachelor’s degrees. The exclusion criteria included students who did not fill out the questionnaires correctly, did not understand Spanish, were positive for COVID-19, and were on pharmacological and/or non-pharmacological therapy for anxiety.

The study population was assessed for suitability using Epidat version 4.2. (Ministry of Health, Xunta de Galicia, Galicia, Spain), which estimated a minimum sample size of 138 university students at a 95% confidence level, with an absolute precision of 1%, and a standard deviation of fear of COVID-19 of 6.04 points (50). The study subjects were selected by a non-probabilistic convenience sampling method and participated voluntarily in the study.

At both universities, the data collection was conducted during class hours. Before that, we had contacted the teachers responsible for the subjects involved so as to minimize the interferences in the correct development of the teaching methodology. The planned place, dates, and times to proceed with the data collection were agreed with the teachers. To maximize the response rate, the study was publicized during breaks between classes and on notice boards in common areas of various university faculties.

The students could complete the questionnaires easily by scanning a QR code of the URL of the Google Form with their smartphones. The questionnaires were created in Google Forms due to the advantages of being flexible, unlimited and free of charge (51). The form contained an informative letter emphasizing the voluntary and anonymous nature of the study, as well as an explicit consent form in which the university students agreed to cooperate and participate in the study.

Finally, data were collected from 1,162 students, and 950 completed all the questionnaire surveys, which satisfied the minimum sample size.

### 2.2. Measurements

#### 2.2.1. Dependent variable: fear of COVID-19 scale (FCV-19S)

The level of emotional reactions of fear toward COVID-19 in individuals was assessed using the COVID-19 Fear Scale (FCV-19S) (30), validated in Spain (50). The scale involves responding to items on a 7-point Likert scale, ranging from ‘strongly disagree’ to ‘totally agree’, with scores ranging from 1 for ‘strongly disagree’ to 5 for ‘totally agree’. The total possible score ranges from 7 to 35, with a higher score indicating a higher level of fear toward COVID-19. The measure showed appropriate internal validity (Cronbach’s alpha: 0.83).

#### 2.2.2. Independent variables

A self-administered survey containing questions concerning the following variables was used to collect the data:

##### 2.2.2.1. Socio-demographic characteristics of Spanish university students

The socio-demographic data collected included the following: gender (women, men), age (18–24 years, 25–35 years, > 35 years), religious belief (yes, no), spiritual practice (yes, no), belief in life after death (yes, no), population of town/city (< 10,000 inhabitants, 10,000–100,000 inhabitants, > 100,000 inhabitants), type of housing (flat without balcony, terrace or courtyard; house without garden or courtyard; flat with balcony, terrace or courtyard; house with garden or courtyard) and number of people sharing the accommodation (not including the participant who answered).

##### 2.2.2.2. Health-related determinants in Spanish university students

The health-related determinants collected included the following: leisure-time physical activity (I do no exercise - I spend my free time almost exclusively sitting down; I occasionally do sports or physical exercise; I do physical exercise several times a month; I do sports or physical exercise several times a week), current smoker (yes, no), frequency of alcohol consumption in the past 12 months (never, less than once a month, monthly, weekly, daily or almost daily) and self-assessed state of health in the past 12 months (very good, good, average, bad, very bad).

##### 2.2.2.3. Variables related to COVID-19 in Spanish university students

The variables related to COVID-19 were collected from a previous study (52) and included the following: living with someone considered to be in a vulnerable group to COVID-19 (yes, no), watching television to stay informed about COVID-19 (little or not at all, only at specific times, most of the day), time spent using the Internet to stay informed about COVID-19 (little or not at all, only at specific times, most of the day), time spent using social networks to stay informed about COVID-19 (little or not at all, only at specific times, most of the day), time spent reading the press (newspapers) to stay informed about COVID-19 (little or not at all, only at specific times, most of the day) (Cronbach’s alpha of 0.62), the probability you think you have of surviving if you become infected with SARS-CoV-2 [5-point Likert scale ranging from 1 (no probability) to 5 (very high probability)], how effective you think preventive measures are to avoid infection with COVID-19 [5-point Likert scale ranging from 1 (not effective at all) to 5 (very effective)], and how satisfied you are with the measures adopted to control the COVID-19 pandemic [5-point Likert scale ranging from 1 (not satisfied at all) to 5 (very satisfied)]. Finally, to prevent the spread of COVID-19, the following items were used (never, rarely, sometimes, nearly always, always): (i) ‘I do not leave the house except to do the shopping or some other essential activity’, (ii) ‘If I have respiratory symptoms, I avoid close contact with other people by staying home’, (iii) ‘I keep a distance of at least 1.5 m from other people’, (iv) ‘When I sneeze or cough, I cover my mouth and nose with my elbow’, (v) ‘I avoid touching my eyes, nose and mouth with my hands’, (vi) ‘I use disposable tissues when sneezing or wiping my nose and throw them away after use’, (vii) ‘I wash my hands frequently’, (viii) ‘I use a mask’, (ix) ‘When I go outside, I mainly stay in outdoor spaces’ and (x) ‘I ventilate closed spaces frequently’ (Cronbach’s alpha of 0.83).

##### 2.2.2.4. General health questionnaire (GHQ-12)

The assessment of common mental disorders was conducted using the 12-item General Health Questionnaire (GHQ-12) (53), which was validated for the Spanish population (54, 55). The GHQ-12 utilizes a Likert-like scale with response options ranging from 0 (‘more than usual’) to 3 (‘much less than usual’). The scoring in the response categories followed the original GHQ method (56), where the first two response options were scored 0 and the last two received a score of 1, resulting in a bimodal score (0–0–1-1). The total score ranged from 0 to 12, with higher scores indicating a greater degree of psychological distress. The measure demonstrated adequate internal validity (Cronbach’s alpha of 0.90).

##### 2.2.2.5. Duke-UNC functional social support questionnaire (DUKE-UNC-11)

The Duke-UNC-11 questionnaire (57), which was validated for use in Spanish populations (58), was utilized to collect information regarding perceived personal social support. The questionnaire comprises 11 items, each scored on a Likert-like scale ranging from 1 (‘much less than I would like’) to 5 (‘as much as I would like’). The total perceived social support score is obtained by adding together the scores of all 11 items, which range from 11 to 55, with higher scores indicating a greater level of perceived social support. Internal validity was adequate (Cronbach’s alpha of 0.90).

##### 2.2.2.6. Death anxiety inventory (DAI)

The level of death anxiety was assessed using the Death Anxiety Inventory (DAI), which was initially developed in Spanish (59). The DAI consists of 20 items, and respondents rated their agreement with each item on a 5-point Likert scale ranging from 1 (‘totally disagree’) to 5 (‘totally agree’). The total possible score ranges from 20 to 100, with higher scores indicating a higher level of death anxiety. The DAI had excellent internal consistency in this sample (Cronbach’s alpha of 0.92).

##### 2.2.2.7. Subjective happiness scale (SHS)

Subjective happiness was assessed using the Subjective Happiness Scale (SHS) (60), which has been validated for use in Spain (61). The scale comprises 4 items presented in a Likert format that measure global subjective happiness through self-rated statements or by comparing oneself to others. The Likert-type scale ranges from 1 (‘not at all’) to 7 (‘to a great extent’), and the total score ranges from 4 to 28 points. Higher scores on the SHS indicate greater levels of global subjective happiness. The scale has an adequate unitary structure and temporal stability, as confirmed in these samples (Cronbach’s alpha of 0.81).

##### 2.2.2.8. Life orientation test-revised (LOT-R)

The Life Orientation Test-Revised (LOT-R) (62, 63), which has been adapted for use in Spanish (64, 65), was used to quantitatively assess the participants’ levels of optimism. The LOT-R comprises ten items, with three items measuring optimism (items 1, 4, and 10), three items measuring pessimism (items 3, 7, and 9), and four neutral filler items (items 2, 5, 6, and 8). Response options are on a Likert scale ranging from 0 (‘strongly disagree’) to 4 (‘strongly agree’), resulting in a total scale range of 0 to 24, which contains both the optimism scale and the inverted pessimism scale. Higher scores on the LOT-R indicate higher levels of optimism. The measure showed appropriate internal validity (Cronbach’s alpha of 0.68).

##### 2.2.2.9. Connor-Davidson resilience scale (CD-RISC)

The level of resilience was measured using the Connor-Davidson Resilience Scale (CD-RISC), a 10-item scale developed by Campbell and Stein (66) and validated in Spain by Notario et al. (67). Each item is rated on a 5-point Likert scale ranging from 0 (‘not true at all’) to 4 (‘true nearly all the time’). The total score ranges from 0 to 40, with higher scores indicating higher levels of resilience. Cronbach’s alpha displayed a good internal consistency (Cronbach’s alpha of 0.84).

##### 2.2.2.10. Athens insomnia scale (AIS-8)

The Athens Insomnia Scale (AIS-8) (68), validated in Spain (69), was used to measure insomnia. The scale consists of 8 items that assess various aspects of sleep difficulty, including sleep induction, night wakings, early morning waking, total sleep time, sleep quality, and the consequences of insomnia the following day, including its effects on functional capacity, well-being, and sleepiness. Responses are rated on a Likert scale from 0 (‘no problem’) to 3 (‘serious problem’). The total score ranges from 0 to 24 points, with higher scores indicating more severe insomnia. The measure showed appropriate internal validity (Cronbach’s alpha of 0.82).

### 2.3. Ethics statement

The study received approval from the clinical research ethics committee (approval number 316, reference 4845).

The study adhered to the principles of good clinical practice and followed the ethical guidelines outlined in the latest version of the Declaration of Helsinki, including the Oviedo agreement, as well as Law 14/2007, dated July 3, concerning Biomedical Research. The confidentiality of the data was strictly maintained at all times by ensuring the anonymity of the data on the database, in compliance with Organic Law 3/2018, dated December 5, on the Protection of Personal Data and guaranteeing digital rights.

### 2.4. Statistical analysis

Descriptive statistics were used to analyze both the categorical variables, including frequencies and percentages, and the quantitative variables, including means and standard deviations. The Kolmogorov–Smirnov test was also used to assess the normality of the variables. Student’s t and ANOVA tests were used to investigate the relationship between FCV-19S scores and the socio-demographic/health-related determinants, variables related to COVID-19 and variables related to the psychological impact of the COVID-19 pandemic, and Pearson’s correlation test was used for correlations between quantitative variables. The variables that demonstrated a statistically significant association with Fear of COVID-19 scores (*p* < 0.05) were later integrated into a multivariable linear regression model. The goodness of fit of the final model was assessed using the adjusted coefficient of determination R^2^. Validation of the collinearity conditions (through analysis of the variance inflation factor), normality, and independence of residuals was confirmed using the normality test and Durbin-Watson test, respectively. The IBM SPSS Statistical package version 26.0.0 (IBM Corp, Armonk, NY, United States), licensed to the University of Seville (Spain), was used to carried out the statistical analysis.

## 3. Results

### 3.1. Descriptive analysis of socio-demographic characteristics and health-related determinants in Spanish university students

We evaluated the data from 950 Spanish university students, mostly aged 18 to 24 years old (92.74%). Most of these students were women (74.11%), did not engage in spiritual practice (70.53%), lived in a town with over 100,000 inhabitants (44.00%), and their self-assessed state of health in the past 12 months was good (55.37%) (Table 1).

**Table 1:** Socio-demographic characteristics and health-related determinants of Spanish university students (*n* = 950).

Variables	Frequency	Percentage
Gender		
Female	704	74.11
Male	246	25.89
Age		
18−24 years	881	92.74
25−35 years	47	4.95
> 35 years	22	2.32
Religious belief		
No	361	38.00
Yes	589	62.00
Spiritual practice		
No	670	70.53
Yes	280	29.47
Believe in life after death		
No	416	43.79
Yes	534	56.21
Population of town/city		
< 10,000 inhabitants	178	18.74
10,000−100,000 inhabitants	354	37.26
> 100,000 inhabitants	418	44.00
Type of housing		
Flat without balcony, terrace or courtyard	122	12.84
House without garden or courtyard	26	2.74
Flat with balcony, terrace or courtyard	427	44.95
House with garden or courtyard	375	39.47
Leisure-time physical activity		
I do no exercise - I spend my free time almost exclusively sitting down	165	17.37
I occasionally do sports or physical exercise	278	29.26
I do physical exercise several times a month	161	16.95
I do sports or physical exercise several times a week	346	36.42
Current smoker		
No	840	88.42
Yes	110	11.58
Frequency of alcohol consumption in the past 12 months		
Never	166	17.47
Less than once a month	300	31.58
Monthly	243	25.58
Weekly	221	23.26
Daily or almost daily	20	2.11
Self-assessed state of health in the past 12 months		
Very good	156	16.42
Good	526	55.37
Average	225	23.68
Bad	38	4.00
Very bad	5	0.53
Variables	Mean	Standard deviation
Number of people sharing the accommodation	2.88	0.98

### 3.2. Descriptive analysis of variables related to COVID-19 in Spanish university students

The majority of participants reported not living with someone considered to be in a group vulnerable to COVID-19 (56.11%), watching television only at specific times to stay informed about COVID-19 (64.95%), using the Internet only at specific times to stay informed about COVID-19 (58.95%), using social networks to stay informed about COVID-19 only at specific times (51.16%), and spending little or no time reading the press (newspapers) to stay informed about COVID-19 (67.58%). In addition to this, the participants felt that the probability of surviving if they became infected with COVID-19 were high (4.49 ± 0.72 points). Moreover, they considered the preventive measures to be moderately effective (3.06 ± 0.88 points) and were moderately satisfied (2.81 ± 0.99 points) with the measures adopted to control the COVID-19 pandemic in Spain.

**Figure 1:**
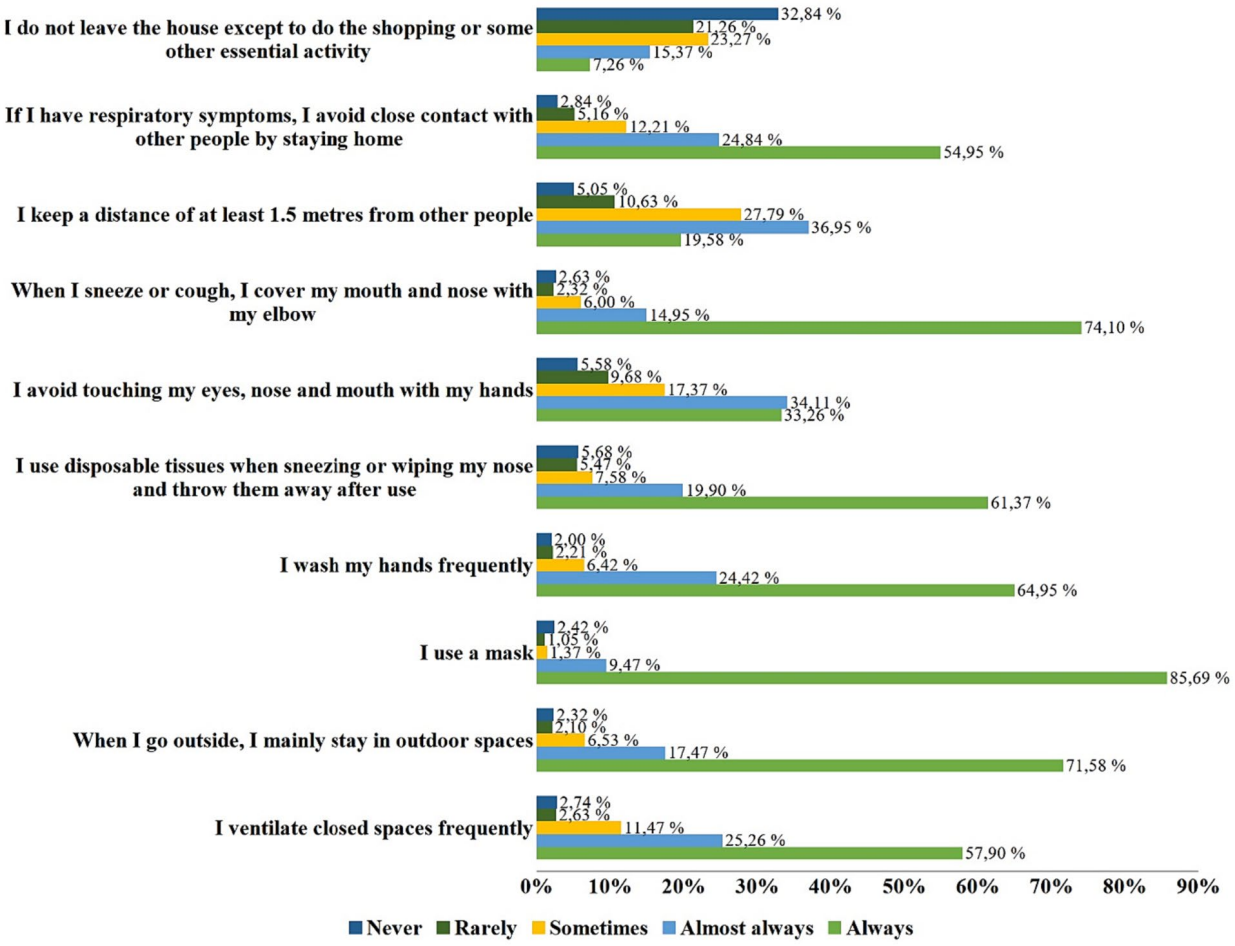
Actions to help preventing the spread of COVID-19 by Spanish university students (*n* = 950).

In terms of actions taken to prevent the spread of COVID-19, the majority of Spanish university students reported always wearing a mask (85.69%), covering their mouth and nose with their elbow when coughing or sneezing (74.10%), and prioritizing outdoor spaces when going outside (71.58%) (Figure 1).

### 3.3. Descriptive and correlational analysis of fear of COVID-19 and variables related to the psychological impact of the COVID-19 pandemic

As shown in Table 2, the average fear of COVID-19 score among Spanish university students was found to be 14.86 ± 5.16 points and was positively correlated with common mental disorders (*r* = 0.680, *p* = 0.036) and death anxiety (*r* = 0.472, *p* < 0.001). On the contrary, fear of COVID-19 was negatively correlated with social support (*r* = −0.081, *p* = 0.012), optimism (*r* = −0.142, *p* < 0.001), resilience (*r* = −0.156, *p* < 0.001) and insomnia (*r* = −0.157, *p* < 0.001).

**Table 2:** Description and correlation between fear of COVID-19 and variables related to the psychological impact of the COVID-19 pandemic in Spanish university students (*n* = 950).

Variables	M (SD) (scores)	FCV-19S r (value of *p*)	GHQ-12 r (value of *p*)	Duke-UNC-11 r (value of *p*)	DAI r (value of *p*)	SHS r (value of *p*)	LOT-R r (value of *p*)	CD-RISC r (value of *p*)	AIS-8 r (value of *p*)
FCV-19S	14.86 (5.16)	1							
GHQ-12	6.14 (1.56)	0.68 (0.036)	1						
Duke-UNC-11	43.00 (9.06)	−0.081 (0.012)	0.053 (0.101)	1					
DAI	52.86 (16.51)	0.472 (<0.001)	0.071 (0.029)	0.006 (0.857)	1				
SHS	18.18 (2.73)	−0.061 (0.060)	−0.002 (0.962)	0.172 (<0.001)	−0.023 (0.488)	1			
LOT-R	14.14 (3.92)	−0.142 (<0.001)	−0.162 (< 0.001)	0.196 (<0.001)	−0.122 (<0.001)	0.349 (<0.001)	1		
CD-RISC	27.50 (6.46)	−0.156 (<0.001)	−0.083 (0.010)	0.183 (<0.001)	−0.162 (<0.001)	0.364 (<0.001)	0.435 (<0.001)	1	
AIS-8	5.91 (3.98)	−0.157 (<0.001)	0.097 (0.003)	−0.256 (<0.001)	0.077 (0.018)	−0.253 (<0.001)	−0.309 (<0.001)	−0.189 (<0.001)	1

FCV-19S, COVID-19 Fear Scale; GHQ-12, General Health Questionnaire; Duke-UNC-11, Functional Social Support Questionnaire; DAI, Death Anxiety Inventory; SHS, Subjective Happiness Scale; LOT-R, Life Orientation Test-Revised; CD-RISC, Connor-Davidson Resilience Scale; AIS-8, Athens Insomnia Scale; M, Mean; SD, Standard deviation; *r*, Pearson’s r.

### 3.4. Relationship of the fear of COVID-19 with socio-demographic characteristics and health-related determinants in Spanish university students

Statistically significant differences in socio-demographic characteristics and health-related determinants among Spanish university students were observed (Table 3). A higher score of fear of COVID-19 was reported among females (*t =* −6.11, *p* < 0.0001), those who lived in a town with less than 10,000 inhabitants (*F* = 1.82, *p* = 0.009), those who practiced a religion (*t =* 0.24, *p* < 0.0001) and believed in life after death (*t =* −1.09, *p* = 0.018), and participants who reported a bad self-assessed state of health in the past 12 months (*F* = 2.07, *p* < 0.0001).

**Table 3:** Comparative analysis of participants’ Fear of COVID-19 scores and socio-demographic characteristics and health-related determinants in Spanish university students (*n* = 950).

Variables	Fear of COVID-19 Scale (score)			
	*M* (SD)	*t* value	*F*	Value of *p*
Gender		−6.11		< 0.001
Female	15.45 (5.09)			
Male	13.16 (5.01)			
Age			1.02	0.518
18−24 years	14.88 (5.17)			
25−35 years	14.91 (4.87)			
> 35 years	13.91 (5.75)			
Religious belief		−2.68		< 0.001
No	14.05 (4.97)			
Yes	15.35 (5.22)			
Spiritual practice		0.24		0.462
No	14.79 (5.21)			
Yes	15.02 (5.06)			
Believe in life after death		−1.09		0.018
No	14.40 (5.05)			
Yes	15.22 (5.22)			
Population of town/city			1.82	0.009
< 10,000 inhabitants	16.06 (5.74)			
10,000−100,000 inhabitants	14.58 (4.91)			
> 100,000 inhabitants	14.58 (5.05)			
Type of housing			1.65	0.859
Flat without balcony, terrace or courtyard	14.64 (5.26)			
House without garden or courtyard	15.58 (6.31)			
Flat with balcony, terrace or courtyard	14.87 (4.96)			
House with garden or courtyard	14.87 (5.28)			
Leisure-time physical activity			0.96	0.524
I do no exercise - I spend my free time almost exclusively sitting down	15.05 (5.25)			
I occasionally do sports or physical exercise	14.92 (5.29)			
I do physical exercise several times a month	15.20 (5.09)			
I do sports or physical exercise several times a week	14.56 (5.05)			
Current smoker		1.28		0.854
No	14.87 (5.22)			
Yes	14.79 (4.68)			
Frequency of alcohol consumption in the past 12 months			1.27	0.099
Never	15.20 (5.60)			
Less than once a month	15.43 (5.54)			
Monthly	14.65 (4.86)			
Weekly	14.03 (4.50)			
Daily or almost daily	15.15 (5.10)			
Self-assessed state of health in the past 12 months			2.07	<0.001
Very good	13.41 (4.95)			
Good	14.62 (4.93)			
Average	16.02 (5.37)			
Bad	17.13 (5.90)			
Very bad	16.00 (5.15)			
Variables	Pearson’s r			Value of *p*
Number of people sharing the accommodation	−0.070			0.777

M, Mean; SD, Standard deviation.

### 3.5. Comparison of the fear of COVID-19 and variables related to COVID-19 in Spanish university students

Fear of COVID-19 was higher in university students who lived with someone considered to be in a group vulnerable to COVID-19 (*t* = −2.24, *p* = 0.022), watched television, used the Internet and social networks most of the day to stay informed about COVID-19 (*F* = 3.41, *p* < 0.0001; *F* = 2.11, *p* = 0.010; *F* = 2.03, *p* < 0.0001, respectively), and spent only a short time reading the press (newspapers) to stay informed about COVID-19 (*F* = 2.03, *p* = 0.040). Lastly, a negative correlation was found between fear of COVID-19 and the probability of the participants believing they could survive if they became infected with COVID-19 (*r* = −0.580, *p* = 0.009; Table 4).

**Table 4:** Comparative analysis of participants’ fear of COVID-19 Scale scores and variables related to COVID-19 in Spanish university students (*n* = 950).

Variables	Fear of COVID-19 Scale (score)			
	M (SD)	*t* value	F	Value of *p*
Living with someone considered to be in a vulnerable group to COVID-19		−2.24		0.022
No	14.49 (5.00)			
Yes	15.33 (5.33)			
Time spent watching television to stay informed about COVID-19			3.41	< 0.001
Little or not at all	13.30 (4.95)			
Only at specific times	15.48 (5.05)			
Most of the day	16.60 (5.87)			
Time spent using the Internet to stay informed about COVID-19			2.11	0.010
Little or not at all	14.08 (5.24)			
Only at specific times	15.21 (5.04)			
Most of the day	15.26 (5.44)			
Time spent using social networks to stay informed about COVID-19			2.03	< 0.001
Little or not at all	13.84 (5.01)			
Only at specific times	15.44 (5.13)			
Most of the day	15.53 (5.33)			
Time spent reading the press (newspapers) to stay informed about COVID-19			1.16	0.040
Little or not at all	14.53 (5.00)			
Only at specific times	15.55 (5.45)			
Most of the day	15.47 (5.10)			
Variables	Pearson’s r			Value of *p*
The probability you think you have of surviving if you become infected with SARS-CoV-2	−0.580			0.009
How effective you think preventive measures are to avoid infection with COVID-19	0.158			0.518
How satisfied you are with the measures adopted to control the COVID-19 pandemic	0.281			0.243

M, Mean; SD, Standard deviation.

### 3.6. Multivariate linear regression model between fear of COVID-19 and independent variables in Spanish university students

The results of the multiple linear regression analyzes are shown in Tables 5, 6 (Adjusted *R*^2^ = 0.35). Fear of COVID-19 was determined by gender (*t* = 3.54, *p* < 0.001), religious beliefs (*t* = 2.00, *p* = 0.04), population of town/city (*t =* −3.33, *t* = −3.41, *p* < 0.01), living with someone considered to belong to a group vulnerable to COVID-19 (*t* = 2.26, *p* = 0.02), watching TV only at specific times (*t =* 5.38, *p* < 0.001) or most of the day (*t =* 3.27, *p* < 0.01) to stay informed about COVID-19, perceived probability of surviving a COVID-19 infection (*t =* −7.62, *p* < 0.001; Table 5), level of death anxiety (*t =* 14.88, *p* < 0.001) and level of insomnia (*t =* 4.43, *p* < 0.001; Table 6).

**Table 5:** Multivariate linear regression model between fear of COVID-19 scores and socio-demographic, health-related determinants and variables related to COVID-19 (*n* = 950).

Variables	Crude analysis				Adjusted analysis^†^			
	*B*	*β*	*t* statistic	Value of *p*	*B*	*β*	*t* statistic	Value of *p*
Gender								
Female	2.29	0.19	6.11	<0.001	1.12	0.10	3.54	< 0.001
Male	Ref.	Ref.			Ref.	Ref.		
Age								
18−24 years	0.97	0.05	0.87	0.38				
25−35 years	1.01	0.04	0.75	0.45				
> 35 years	Ref.	Ref.						
Religious belief								
No	Ref.	Ref.		< 0.001	Ref.	Ref.		0.04
Yes	1.30	0.12	3.80		0.57	0.05	2.00	
Spiritual practice								
No	Ref.	Ref.		0.54				
Yes	0.22	0.02	0.61					
Believe in life after death								
No	Ref.	Ref.		0.02				
Yes	0.81	0.08	2.41					
Population of town/city								
< 10,000 inhabitants	Ref.	Ref.			Ref.	Ref.		
10,000−100,000 inhabitants	–1.48	–0.14	−3.14	<0.01	–1.29	–0.12	−3.33	<0.01
> 100,000 inhabitants	–1.47	–0.13	−3.22	<0.01	–1.28	–0.11	−3.41	<0.01
Type of housing								
Flat without balcony, terrace or courtyard	Ref.	Ref.						
House without garden or courtyard	0.94	0.03	0.84	0.40				
Flat with balcony, terrace or courtyard	0.23	0.02	0.43	0.67				
House with garden or courtyard	0.24	0.02	0.44	0.66				
Leisure-time physical activity								
I do no exercise - I spend my free time almost exclusively sitting down	Ref.	Ref.						
I occasionally do sports or physical exercise	–0.31	–0.01	−0.26	0.80				
I do physical exercise several times a month	–0.16	0.01	−0.27	0.78				
I do sports or physical exercise several times a week	–0.49	–0.05	−0.99	0.32				
Current smoker								
No	Ref.	Ref.		0.88				
Yes	–0.08	–0.005	−0.15					
Frequency of alcohol consumption in the past 12 months								
Never	Ref.	Ref.						
Less than once a month	0.23	0.02		0.65				
Monthly	–0.55	–0.05		0.29				
Weekly	–1.17	–0.10		0.03				
Daily or almost daily	–0.06	–0.002		0.96				
Self-assessed state of health in the past 12 months								
Very good	Ref.	Ref.						
Good	1.21	0.12	2.61	<0.01				
Average	2.61	0.22	4.93	<0.001				
Bad	3.72	0.14	4.05	<0.001				
Very bad	2.59	0.04	1.12	0.26				
Number of people sharing the accommodation	–0.36	–0.07	−2.12	0.03				
Living with someone considered to be in a vulnerable group to COVID-19								
No	Ref.	Ref.		0.01	Ref.	Ref.		0.02
Yes	0.84	0.08	2.49		0.63	0.06	2.26	
Time spent watching television to stay informed about COVID-19								
Little or not at all	Ref.	Ref.			Ref.	Ref.		
Only at specific times	2.18	0.20	6.06	<0.001	1.63	0.15	5.38	<0.001
Most of the day	3.30	0.13	3.95	<0.001	2.28	0.09	3.27	<0.01
Time spent using the Internet to stay informed about COVID-19								
Little or not at all	Ref.	Ref.						
Only at specific times	1.13	0.11	3.06	<0.01				
Most of the day	1.18	0.07	1.92	0.06				
Time spent using social networks to stay informed about COVID-19								
Little or not at all	Ref.	Ref.						
Only at specific times	1.60	0.16	4.46	<0.001				
Most of the day	1.69	0.11	3.08	<0.01				
Time spent reading the press (newspapers) to stay informed about COVID-19								
Little or not at all	Ref.	Ref.						
Only at specific times	1.02	0.09	2.81	<0.01				
Most of the day	0.94	0.03	0.79	0.43				
The probability you think you have of surviving if you become infected with SARS-CoV-2	–2.32	–0.32	−10.48	<0.001	–1.49	–0.21	−7.62	<0.001
How effective you think preventive measures are to avoid infection with COVID-19	–0.25	–0.04	−1.31	0.19				
How satisfied you are with the measures adopted to control the COVID-19 pandemic	–0.25	–0.05	−1.45	0.15				

^†^Adjusted for variables related to the psychological impact of the COVID-19 pandemic; B = Estimated parameter; Ref.: Reference; Adjusted R-Squared = 0.35; *F* = 51.24; *p* < 0.001.

**Table 6:** Multivariate linear regression model between fear of COVID-19 scores and variables related to the psychological impact of the COVID-19 pandemic of Spanish university students (*n* = 950).

Variables	Crude analysis				Adjusted analysis^†^			
	*B*	*β*	*t* statistic	*value of p*	*B*	*β*	*t* statistic	*value of p*
Common mental disorders	0.19	0.06	1.73	0.08				
Perceived social support	–0.04	–0.08	−2.39	0.02				
Death anxiety	0.15	0.48	16.75	<0.001	0.13	0.40	14.88	< 0.001
Subjective happiness	–0.11	–0.06	−1.78	0.08				
Optimism	–0.19	–0.14	−4.43	<0.001				
Resilience	–0.10	–0.15	−4.68	<0.001				
Insomnia	0.23	0.18	5.54	<0.001	0.16	0.12	4.43	<0.001

^†^Adjusted for socio-demographic, health-related determinants and variables related to COVID-19; B = Estimated parameter; Ref.: Reference; Adjusted R-Squared = 0.35; F = 51.24; *p* < 0.001.

## 4. Discussion

### 4.1. Main findings

The present study presents a multidisciplinary approach to examine the relationship between fear of COVID-19 and multiple factors that may influence the psychological impact of the COVID-19 pandemic on Spanish university students. Overall, the study found that the average fear of COVID-19 score was 14.86 ± 5.16 points. Several factors were identified as predictors of fear of COVID-19, including being female, holding religious beliefs, residing in towns/cities with over 10,000 inhabitants, living with someone vulnerable to COVID-19, and watching TV to stay informed about COVID-19 either at specific times or for most of the day. Additionally, the higher the participants’ perceived probability of surviving a COVID-19 infection, the lower their fear of COVID-19 score. However, the fear of COVID-19 score increased with every one-point increase in the level of death anxiety and insomnia.

According to our results, the level of fear of COVID-19 among Spanish university students was found to be lower than that reported in other studies conducted in the same context in Spain (25, 70, 71). In contrast, other countries have reported higher average scores of fear of COVID-19, including China (27), Pakistan (28), Russia and Belarus (29) and Turkey (26). Spain was one of the countries in the world most affected by the first wave of the COVID-19 pandemic (72–74). The measures implemented by the Spanish government, autonomous communities and Spanish universities to ensure the safety and well-being of students during the COVID-19 pandemic (75, 76) may have contributed to reducing fear of COVID-19 in this population group. In fact, our study also found that the majority of participants reported engaging in preventive practices such as wearing masks, covering their mouth and nose with their elbow when coughing or sneezing, and always prioritizing outdoor spaces when going outside. In that context, measures such as limiting face-to-face attendance at universities (77), promoting online classes (78), and implementing health safety protocols (79) may have contributed to creating a sense of safety and confidence among students (80).

Fear of COVID-19 may have affected women and men differently (81), with women having a higher perceived risk of infection (82). Various studies have shown that women experienced higher levels of stress, anxiety, and depression related to the COVID-19 pandemic compared to men (83–85). It is also important to note that women often take on caregiving roles (86, 87) and work as front-line health-care workers (88), which put them at greater risk and vulnerability during COVID-19 pandemic (89).

One of the best-known coping strategies to deal with the negative effects that the COVID-19 pandemic had on the mental health of the population was having religious beliefs, attitudes, or practices (90–94). Nevertheless, our study participants who believed in a religion showed a higher score for fear of COVID-19, in line with other studies (95–97). According to Krok et al. (98), the fear of COVID-19 also intensified the effect of religiosity on meaning-making, leading individuals to seek religious activities for emotional support and coping strategies.

According to our results, university students who lived in a town/city with over 10,000 inhabitants had higher fear of COVID-19 scores, in agreement with other studies (99, 100). This may be because people living in urban areas may be more exposed to the virus due to the higher population density and more frequent social interactions (101).

We also found that university students who lived with someone considered to be in a high-risk group for COVID-19 experienced a significantly greater fear of COVID-19, in line with other studies (102, 103). It is likely that people experienced higher levels of anxiety due to the fear of infecting their sick relatives as well as from the fear of contracting SARS-CoV-2 coronavirus themselves (104). Watching TV to stay informed about COVID-19 was identified as another factor that contributed to the greater fear of COVID-19 among study participants. This finding is consistent with the idea that the coverage of COVID-19 in the mainstream media focused predominantly on negative issues (105), disseminating uncertainties about the virus, the global spread of the pandemic (106), the increasing number of cases and fatalities (107), government policies (108), and the rising demand for healthcare (109), all of which had psychological implications.

Finally, university students reported feeling less fear of COVID-19 if they believed they could survive the disease, whether they were already infected or might become infected in the future, in agreement with Tusey et al. (110). In fact, participants in the study by Mousavi et al. (111), who believed they had a low probability of surviving if infected with COVID-19 were more likely to practice preventive behavior. Considering that a significant majority of the participants in the present study were under 25 years of age, this is likely to be due to the scientific literature reporting high SARS-CoV-2 transmissibility but relatively low mortality rates in this age range (112–114). Nevertheless, survivors who had COVID-19 perceived the disease as a factor affecting their existence and reported having a fear of death (115–118). In fact, it is not surprising that death anxiety arises when individuals face the threat of death due to either experiencing or fearing COVID-19 (119). In this case, the effective utilization of mindfulness and coping strategies can assist individuals in managing stressful situations, decreasing negative emotions (120) and improving sleep quality (121). Fear plays a determinant factor in modifying normal sleep patterns (122, 123), and so the connection between the fear of COVID-19 and sleep problems could be linked to concerns about the contagious nature of the disease (124), as supported by other studies (125, 126). Nevertheless, when Cerqueira et al. (127) asked university students about the quality of their sleep in the COVID-19 pandemic, 55.7% described it as either ‘very good’ or ‘good in general’. In addition, Wright et al. (128) reported that during the lockdown period, time in bed increased by around 30 min on weekdays and 24 min at weekends, sleep timing regularity improved by approximately 12 min, and university students extended the duration of their sleep by about 50 min on weekdays and approximately 25 min at weekends, thereby reducing social jetlag. Leone et al. (129) attributed the improvement in weekday sleep duration and the reduction in social jetlag to a plausible explanation based on lifestyle changes associated with weaker social cues, such as work and class schedules becoming more flexible, delayed, or even absent. t.

### 4.2. Strengths and limitations

One of the major strengths of this study was its multidisciplinary approach, which explored the relationship between fear of COVID-19 and various factors that may have affected the psychological impact of the pandemic on Spanish university students, using a considerable number of validated questionnaires. Nevertheless, the study has several limitations. Firstly, its cross-sectional design means that no causal relationships can be established. Secondly, the study was conducted from December 2020 to December 2021, and therefore further research is needed to investigate how levels of fear of COVID-19 may have changed over time. Thirdly, the use of questionnaires as a data collection method is subject to the participants’ truthfulness in responding. For example, using self-report measures may not reflect people’s real opinions and feelings due to the demands of social desirability (130). Lastly, the study’s non-probabilistic convenience sample of university students from the southern region of Spain limits the generalizability of its findings.

### 4.3. Implications for research and practice

This study highlights the significance of fear of COVID-19 and its impact on the mental health of university students. Previous research has demonstrated that a significant proportion of Spanish university students suffer from mental health disorders (131), and that the COVID-19 pandemic exacerbated negative mental health outcomes (132). These findings can help to identify specific factors that contribute to the fear of COVID-19 and enable us to develop coping strategies to alleviate the stress caused by the pandemic. They may also help policymakers and health professionals in devising suitable strategies to address the pandemic’s long-term effects on mental health, for example, by emphasizing the need to enable universities to make decisions regarding changes to the curriculum and assessment methods that align with students’ well-being and mental health expectations (133). This could include flexible academic schedules, reduced workloads during future pandemics, and the option of distance-learning to accommodate students’ needs during a pandemic (134). Furthermore, the study suggests that universities may be an ideal environment for implementing health promotion programs focusing on mental health and well-being (135), as they allow students to develop coping skills and better understand how to manage fear during a pandemic.

## 5. Conclusion

A score of 14.86 ± 5.16 points was identified as the average level of fear of COVID-19 among university students in Spain. The main predictors of fear of COVID-19 are being female, having religious beliefs, living in towns/cities with over 10,000 inhabitants, living with someone vulnerable to COVID-19, watching TV to stay informed about COVID-19, believing in a low probability of survival if infected with COVID-19, and having a higher level of death anxiety and insomnia. The findings of this study, which identify numerous factors related to fear of COVID-19, can aid policymakers and health professionals in developing appropriate strategies to address the pandemic’s long-term effects on mental health. In addition, these findings can facilitate future research on coping strategies for university students facing the stress caused by a pandemic.

## Data availability statement

The raw data supporting the conclusions of this article will be made available by the authors, without undue reservation.

## Ethics statement

The studies involving humans were approved by Ethical Committee for Clinical Research of Cordoba (Spain) (approval number 316, reference 4845). The studies were conducted in accordance with the local legislation and institutional requirements. The participants provided their written informed consent to participate in this study.

## Author contributions

JC: conceptualization, methodology, and writing–original draft. JC and SP: data curation, data analysis, resources, visualization, project administration, methodology, and writing–reviewing and editing. SP: supervision. All authors contributed to the article and approved the submitted version.
